# Impact of dietary organic acids and botanicals on intestinal integrity and inflammation in weaned pigs

**DOI:** 10.1186/s12917-015-0410-0

**Published:** 2015-04-16

**Authors:** Ester Grilli, Benedetta Tugnoli, Jade L Passey, Chad H Stahl, Andrea Piva, Adam J Moeser

**Affiliations:** DIMEVET, University of Bologna, Ozzano Emilia, 40064 Bologna, Italy; University of Surrey, Guildford, Surrey GU2 7XH UK; North Carolina State University, Department of Population Health and Pathobiology, Raleigh, NC 27695 USA; Laboratory of Developmental Nutrition, College of Agriculture and Life Sciences, North Carolina State University, Raleigh, NC 27695, USA; Center for Comparative Medicine and Translational Research (CCMTR), North Carolina State University, College of Veterinary Medicine, Raleigh, NC 27695 USA

**Keywords:** Organic acids, Pure botanicals, Weaning piglet, Inflammatory cytokines, Intestinal barrier

## Abstract

**Background:**

Organic acids, such as citric and sorbic acid, and pure plant-derived constituents, like monoterpens and aldehydes, have a long history of use in pig feeding as alternatives to antibiotic growth promoters. However, their effects on the intestinal barrier function and inflammation have never been investigated. Therefore, aim of this study was to assess the impact of a microencapsulated mixture of citric acid and sorbic acid (OA) and pure botanicals, namely thymol and vanillin, (PB) on the intestinal integrity and functionality of weaned pigs and *in vitro* on Caco-2 cells. In the first study 20 piglets were divided in 2 groups and received either a basal diet or the basal diet supplemented with OA + PB (5 g/kg) for 2 weeks post-weaning at the end of which ileum and jejunum samples were collected for Ussing chambers analysis of trans-epithelial electrical resistance (TER), intermittent short-circuit current (*I*_SC_), and dextran flux. Scrapings of ileum mucosa were also collected for cytokine analysis (n = 6). In the second study we measured the effect of these compounds directly on TER and permeability of Caco-2 monolayers treated with either 0.2 or 1 g/l of OA + PB.

**Results:**

Pigs fed with OA + PB tended to have reduced *I*_SC_ in the ileum (*P* = 0.07) and the ileal gene expression of IL-12, TGF-β, and IL-6 was down regulated. In the *in vitro* study on Caco-2 cells, TER was increased by the supplementation of 0.2 g/l at 4, 6, and 14 days of the experiment, whereas 1 g/l increased TER at 10 and 12 days of treatment (*P* < 0.05). Dextran flux was not significantly affected though a decrease was observed at 7 and 14 days (*P* = 0.10 and *P* = 0.09, respectively).

**Conclusions:**

Overall, considering the results from both experiments, OA + PB improved the maturation of the intestinal mucosa by modulating the local and systemic inflammatory pressure ultimately resulting in a less permeable intestine, and eventually improving the growth of piglets prematurely weaned.

## Background

The intestinal barrier consists of a single layer of enterocytes and connecting inter-epithelial tight junctions which are responsible of regulating the para-cellular flux of solutes and macromolecules [1]. When inflammation occurs, like at weaning, the epithelial barrier and tight junctions functionality are impaired and the ability of the enterocyte to absorb nutrients decreases. This eventually results in delayed growth performance associated with an increased susceptibility to infections [2,3]. The inflammation-driven impairment of the intestinal barrier function at weaning also has strong repercussions at later age as at 9 weeks pigs still suffer the intestinal maturation gap provoked by early weaning [4]. Therefore, the importance of maintaining a healthy mucosa and a selective intestinal barrier in this critical phase appears strategic for long-life pig performance.

Organic acids and plant derivatives have a long history of use in animal nutrition as alternative -or in addition- to conventional antibiotic growth promoters and their mode of action as growth enhancers has been generally associated with a decreased load of pathogenic bacteria in the gut [5]. More specifically, sorbic acid, a long chain unsaturated fatty acid, is a strong antimicrobial at relatively neutral pH, and exerts its action by inhibiting the microbial enzymatic apparatus and nutrient transport system [6]. Citric acid, instead, has a moderate antimicrobial action, as it can be metabolized by the bacterial cell itself, but by entering the TCA-cycle may serve as energy supply for the host as well. Nonetheless, citric acid is one of the most studied organic acid in pig weaning diets and its efficacy *in vivo* has been extensively proven [5]. On the other side, plant-derived compounds include a large variety of molecules with different chemical properties. In particular, monterpens, such as thymol, are proposed as bacterial membrane permeabilizers, or pore-forming agents, that allow ion leakage and membrane potential disruption [7]. Along with aldehydes, such as vanillin, they are also used as flavoring agents to improve feed palatability and digestibility [8]. Moreover, these compounds have also anti-oxidant and anti-inflammatory properties [9,10].

The unique combination of these molecules in a slow-release matrix allowed in the past to consistently measure improved performance of weaning piglets, and also of other animal species [11-13], but a link between growth enhancement and gut health has never been clarified so far.

The aim of this study was to investigate whether this specific combination of compounds has an impact on intestinal health and mucosa barrier function at weaning. Moreover, we wanted also to determine whether the compounds could have a direct effect - not-microflora mediated- on intestinal epithelial cells. To respond these questions, we performed 2 experiments: the first was designed to assess the impact of microencapsulated sorbic, citric acid, thymol and vanillin, on measures of intestinal inflammation and intestinal integrity parameters in healthy pigs weaned at an early age; in the second experiment, instead, as we wanted to exclude the interference of the microbiota in the response of the mucosa to organic acids and botanicals, we assessed the effect of these compounds directly on trans-epithelial resistance and permeability of Caco-2 cell cultures.

## Methods

### *In vivo* assessment on weaned pigs

#### Experimental procedure

The study was approved by the North Carolina State University Institutional Animal Care and Use Committee. Twenty piglets (commercial hybrids) were weaned at 18 days of age and divided in 2 groups (*n* = 10) receiving either a basal diet (control group) or the basal diet supplemented with microencapsulated organic acids and pure botanicals for 2 weeks (**OA + PB**, 5 g/kg of feed, providing 16.7% sorbic acid, 25% citric acid, 1.7% thymol and 1% vanillin in a matrix of hydrogenated fats; Aviplus®-S, Vetagro SpA, Reggio Emilia, Italy; US patent # 7,258,880; EU patent # 1-391-155B1; CA patent # 2,433,484). The control diet contained 2.5% plasma protein (APC, Ankeny, IA), 35.5% SBM (48% CP), 58.7% corn (US #2), 1.65% dicalcium phosphate, 1% limestone, and 1% corn oil, along with a vitamin and mineral premix to meet or exceed the nutrient requirements for this size/age of pig [14]. No antibiotics were added to this diet. Individual body weights were recorded initially (d0), at day 7 (d7) and at day 14 (d14), and average daily gain (**ADG**) was calculated. Blood samples were collected at d0 and at every 7 days by venipuncture using tubes without anticoagulant, and sera samples were stored at −20°C until analysis of inflammatory cytokines.

At the completion of the study 6 pigs per group were selected for sacrifice, sample collection and analysis. Piglets were killed by penetrating captive bolt followed by exsanguination. Segments of jejunum and ileum were collected and immediately prepared for Ussing chamber analysis. Ileal mucosa scrapings were collected, snap-frozen in liquid N_2_ and stored at −80°C until gene and protein expression analysis.

#### Ussing chamber analysis

Ussing chamber experiments were conducted according to Smith et al. [4]. Segments of jejunum and ileum were collected from the pig, and the mucosa was stripped from the seromuscular layer in oxygenated (95% O_2_-5% CO_2_) Ringer solution (in mmol/l: 154 Na^+^, 6.3 K^+^, 137 Cl^−^, 0.3 H_2_PO_4_, 1.2 Ca^2+^, 0.7 Mg^2+^, 24 HCO_3_^−^ pH 7.4). Tissues were then mounted in 1.13-cm^2^ aperture Ussing chambers and bathed on the serosal and mucosal sides with 10 mL of Ringer solution. The serosal bathing solution contained 10 mm glucose, which was osmotically balanced on the mucosal side with 10 mm mannitol. Bathing solutions were oxygenated (95% O_2_-5% CO_2_) and circulated in water-jacketed reservoirs maintained at 37°C. The spontaneous potential difference (**PD**) was measured using Ringer-agar bridges connected to calomel electrodes, and the PD was short-circuited through Ag-AgCl electrodes using a voltage clamp that corrected for fluid resistance. Tissues were maintained in the short-circuited state, except for short intervals to record the open-circuit PD. Trans-epithelial electrical resistance (**TER**; Ω · cm^2^) was calculated from the spontaneous PD and short-circuit current (***I***_**SC**_), as previously described [15]. After a 30-min equilibration period on Ussing chambers, TER was recorded at 15-min intervals over a 1-h period and then averaged to derive the basal TER values for a given animal.

Mucosal permeability was assessed measuring mucosal-to-serosal fluxes of dextran, according to Moeser et al. [16]. FITC-dextran 4 kDa (**FD4**) was added to the serosal bathing reservoir of Ussing chambers at a concentration of 100 mg/ml and the flux to the serosal compartment was measured. After a 30-min equilibration period, standards were taken from the serosal side of each chamber. Samples (0.5 ml) were collected from the serosal chambers at 30-min intervals over a 90-min period. The quantity of FD4 was established by measuring the fluorescence in serosal reservoir fluid samples in a fluorescence plate reader at 540 nm. Data are presented as the rate of FD4 flux (mg/cm^2^/min) over a 90-min period.

#### ELISA quantification of inflammatory cytokines

Protein levels of interferon- γ (**IFN-γ**), transforming growth factor-β (**TGF-β**), tumor necrosis factor- α (**TNF-α**), interleukin-6 (**IL-6**), interleukin-10 (**IL-10**), interleukin-12 (**IL-12**) were determined both in sera samples and in ileal samples using commercial ELISA kits specific for porcine cytokines (R&D Systems, Minneapolis, MN). Sera samples were prepared according to manufacturer’s instructions. Ileal mucosa scrapings were lysed in lysis buffer (10 mm-Tris, 1 mm-EDTA, 0.5%-Triton X100) and homogenized using a handheld rotor-stator homogenizer before ELISA analysis. Analyses were performed according to manufacturer’s instructions. Results refer to picograms of cytokine per 100 mg of tissue (pg/100 mg).

#### Inflammatory cytokines, SGLT-1 and CFTR gene expression analysis

Ileal mucosa scrapings were disrupted by grinding in liquid N_2_ with mortar and pestle, then homogenized using a handheld rotor-stator homogenizer. Total RNA was isolated using RNeasy Mini Kit (Qiagen, Hilden, Germany) according to the manufacturer’s instructions. Genomic DNA contamination was removed by treatment with deoxyribonuclease (Ambion DNA-free kit, Life Technologies, Grand Island, NY). RNA yield and quality were determined spectrophotometrically using A_260_ and A_280_ nm measurements. All samples showed A_260_:A_280_ ratio of 1.9–2.0. A total of 1 μg of RNA was reverse transcribed with Superscript III (Invitrogen Life Technologies, Grand Island, NY) according to the manufacturer’s instructions. The cDNA samples were then treated with RNase H (Invitrogen Life Technologies, Grand Island, NY) to ensure the removal of residual RNA, then cDNA was quantified with Quant-iT Oligreen ssDNA Assay (Life Technologies, Grand Island, NY) in order to normalize the quantity of cDNA template used for amplification by real-time PCR. Relative quantities of the transcripts of interest were determined by semi-quantitative real-time PCR (My iQ Single Color Real-Time PCR Detection System and SybrGreen Supermix, Bio-Rad Laboratories, Hercules, CA). Thermocycling protocol included initial denaturation for 3 min at 95°C, then 40 cycles of 20 s of denaturation at 95°C followed by 20 s of annealing and extension at 60°C. Following amplification, all samples were subjected to a melt curve analysis to exclude presence of non-specific products. Gene expression was normalized using two housekeeping genes coding for portions of porcine ribosomal subunit 60S, such as ribosomal protein L35 (**RPL35**) and ribosomal protein L4 (**RPL4**). Average C_T_ was determined for each gene of interest (**GOI**), and geometric average was calculated for housekeeping genes (**HKs**), assuming C_T_ as number of cycles needed to reach a fixed arbitrary threshold. Delta C_T_ was calculated as C_T_ GOI − C_T_ HKs, then a modification of the 2^–ΔΔC^_T_ method was used to analyze the relative expression (fold changes), calculated relative to the control group [17].

The sequences, expected product length, accession number in the GenBank database and references of porcine primers are shown in Table 1. Primer oligonucleotides for IL-12, TNF-α, TGF-β, sodium/glucose co-transporter 1 (**SGLT-1**), and cystic fibrosis transmembrane conductance regulator (**CFTR**) were designed using PrimerQuest software (IDT, Integrated DNA Technologies, www.idtdna.com). Primers were obtained from IDT, Integrated DNA Technologies (www.idtdna.com).

**Table 1:** **Primers used for gene expression analysis**

**Gene**	**Primer sequence (F and R; 5′ → 3′)**	**Product length (bp)**	**Product Tm (°C)**	**GenBank accession No.**	**Reference**
**IL-6**	F: AGCAAGGAGGTACTGGCAGAAAACAAC	110	83.5	[NM_214399.1]	[33]
	R: GTGGTGATTCTCATCAAGCAGGTCTCC				
**IL-10**	F: CGGCGCTGTCATCAATTTCTG	89	85	[NM_214041.1]	[34]
	R: CCCCTCTCTTGGAGCTTGCTA				
**IL-12**	F: GCCCAGGAATGTTCAAATGCCTCA	199	85.5	[NM_213993.1]	Present study
	R: AGGCAACTCTCATTCGTGGCTAGT				
**TNF-α**	F: GCCCACGTTGTAGCCAATGTCAAA	99	87	[NM_214022.1]	Present study
	R: GTTGTCTTTCAGCTTCACGCCGTT				
**TGF-β**	F: AGGCCGTACTGGCTCTTTACAACA	134	83.5	[NM_214015.1]	Present study
	R: TTGGTTGCCGCTTTCCACCATTAG				
**IFN-γ**	F: GGCCATTCAAAGGAGCATGGATGT	149	83.5	[NM_213948.1]	Present study
	R: TGAGTTCACTGATGGCTTTGCGCT				
**SGLT-1**	F: TGCTGTTTCCAGATGATGTGGGCT	198	90.5	[NM_001012297.1]	Present study
	R: TGCTGCTGCTGTTAAAGATGGACG				
**CFTR**	F: GAAGAACTGGATCAGGGAAGAG	97	84.5	[NM_001104950.1]	Present study
	R: GAATCCCAAGACACACCATCTA				
**RPL35**	F: AACCAGACCCAGAAAGAGAAC	146	87.5	[NM_214326.2]	[35]
	R: TTCCGCTGCTGCTTCTTG				
**RPL4**	F: CAAGAGTAACTACAACCTTC	122	84	[XM_003121741.3]	[35]
	R: GAACTCTACGATGAATCTTC				

*Abbreviations*: *F* = forward primer, *R* = reverse primer, *Tm* = melting temperature, *IL-6* = interleukin-6, *IL-10* = interleukin-10, *IL-12* = interleukin-12, *TNF-α* = tumor necrosis factor-α, *TGF-β* = transforming growth factor-β, *IFN-γ* = interferon-γ, *SGLT-1* = sodium/glucose co-transporter-1, *CFTR* = cystic fibrosis transmembrane conductance regulator, *RPL35* = ribosomal protein L35, *RPL4* = ribosomal protein L4.

### In vitro assessment on Caco-2 cells

Cells were grown and transplated according to the procedure by Collington et al. [18]. Briefly, Caco-2 cells were grown in Dulbecco’s Modified Eagle Medium (**DMEM**) supplemented with 1% non-essential amino acids, 2 mmol/l L-glutamine, and 10% fetal calf serum. Cells were maintained in a humidified atmosphere of 5% CO_2_ in air at 37°C. Single-cell suspensions were obtained from confluent serial cultures by incubation with 0.25% trypsin and 0.02% EDTA in Ca^2+^- and Mg^2+^-free Hanks balanced salt solution. Caco-2 cells were seeded at a density of 5 × 10^4^ cells/cm^2^ on to Transwell polycarbonate microporous cell culture inserts (24.5 mm diameter, 0.4 μm pore; Costar Ltd, High Wycombe, Bucks, UK); monolayers were used about 1 month after seeding, by which time trans-epithelial electrical resistance (TER) was stable (~150 Ω∙cm^2^) and cells exhibited a morphologically distinct apical brush border. After, cells were cultured with DMEM containing OA + PB at 0.2, or 1 g/l for 14 days.

For measurement of trans-epithelial electrical parameters, chopstick electrodes were used to measure TER at 0, 4, 6, 10, 12, and 14 days, whereas mucosal-to-serosal fluxes of dextran were measured at 7 and 14 days by applying FD4 (100 mg/ml) on the apical well of the transwell and collecting basal media after 24 h for measurements of FD4 fluorescence.

All cell culture media, supplements, and reagents were purchased from Sigma (Sigma- Aldrich, St. Louis, MO).

#### Statistical analysis

Data were analyzed using a two-tail Student’s *t*-test with the GLM procedure of SAS (SAS Institute Inc., release 9.2, Cary, NC). Gene expression and cytokine data were analyzed without covariate. Collection day was used as a covariate for the Ussing chamber data. Initial body weight (**BW**) was used as a covariate for growth performance data. Caco-2 cells statistical analysis was conducted with ANOVA repeated measures for TER and with one-way ANOVA for dextran fluxes followed by Bonferroni and Dunnett post-test, respectively, to compare treated groups with control. In the *in vivo* study the animal was the experimental unit (n = 10 for growth performance; n = 6 for Ussing chamber data, gene expression, and cytokine profiling), whereas in the Caco-2 study the experimental unit was the well (n = 7). Differences were considered significant at *P* < 0.05, trends at 0.05 < *P* ≤ 0.10.

## Results

### In vivo assessment on weaned pigs

#### Growth performance and Ussing chamber analyses

**Figure 1:**
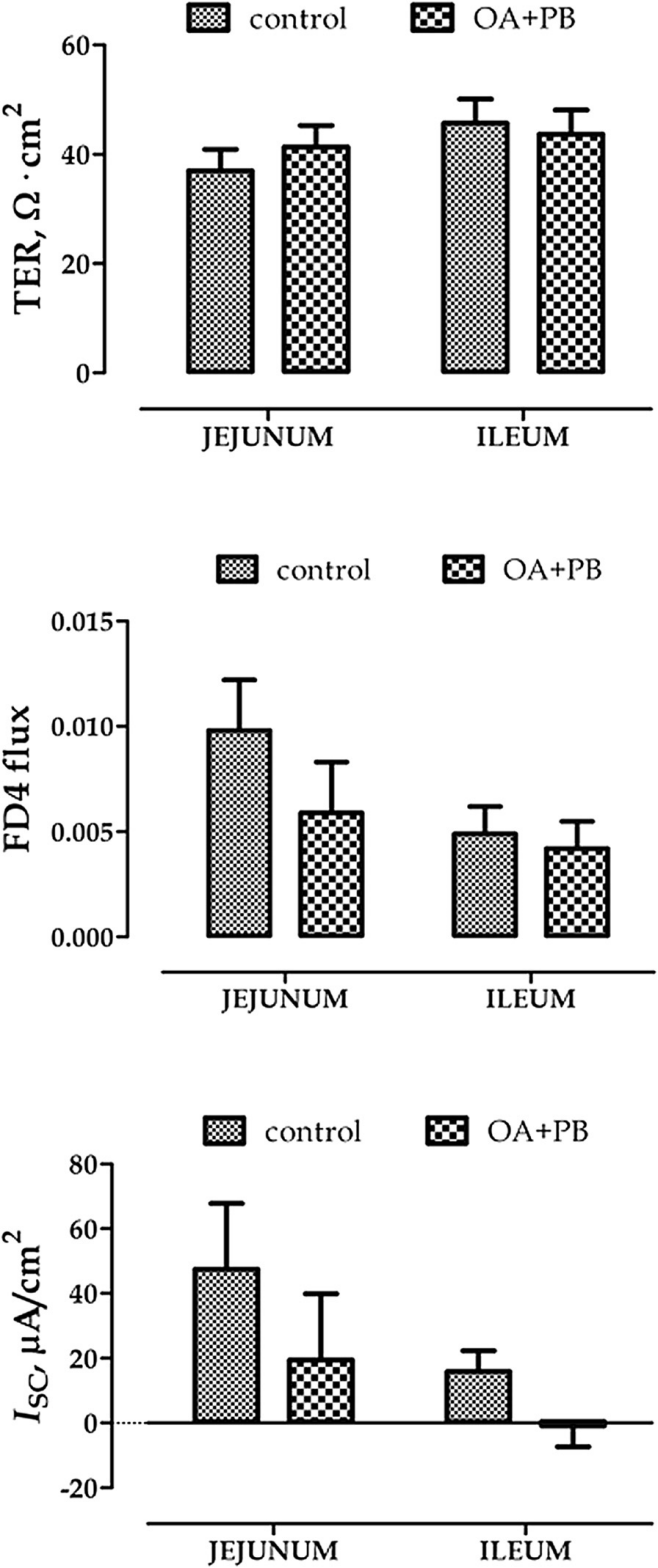
Ussing chamber analysis of jejunum and ileum. Values are least square means (*n* = 6) and SEM represented by vertical bars. TER = trans-epithelial electrical resistance (Ω · cm^2^); FD4 flux = mucosal-to-serosal FITC-dextran flux (mg/cm^2^/min); *I*_SC_ = short-circuit current (μA/cm^2^). Control = basal diet; OA + PB = basal diet + mixture of organic acids and pure botanicals at 5 g/kg.

Growth performance is summarized in Table 2. Treated pigs had higher BW compared with controls at d14 (9.13 kg versus 8.45 kg; *P* < 0.05) and greater ADG during the second week of the study and in the overall period (*P* < 0.05). Results from Ussing chamber analysis are reported in Figure 1. In OA + PB group intermittent short-circuit current (*I*_SC_) tended to be reduced in the ileum (*P* = 0.07), whereas the other parameters were unaffected.

**Table 2:** **Growth performance**

	**Treatment**			
**Item**	**Control**	**OA + PB**	**SEM**	***P***
BW, kg				
d0	7.08	7.15	0.07	0.91
d7	8.08	8.01	0.08	0.57
d14	8.46	9.13	0.21	0.049
ADG, g/d				
0 to 7d	136.8	127.4	11.7	0.59
7 to 14d	191.6	286.9	29.6	0.049
0 to 14d	95.9	143.6	14.8	0.049

Control = basal diet; OA + PB = basal diet + mixture of organic acids and pure botanicals at 5 g/kg.

BW = body weight; ADG = average daily gain.

#### Inflammatory cytokines, SGLT-1 and CFTR gene expression analysis

**Figure 2:**
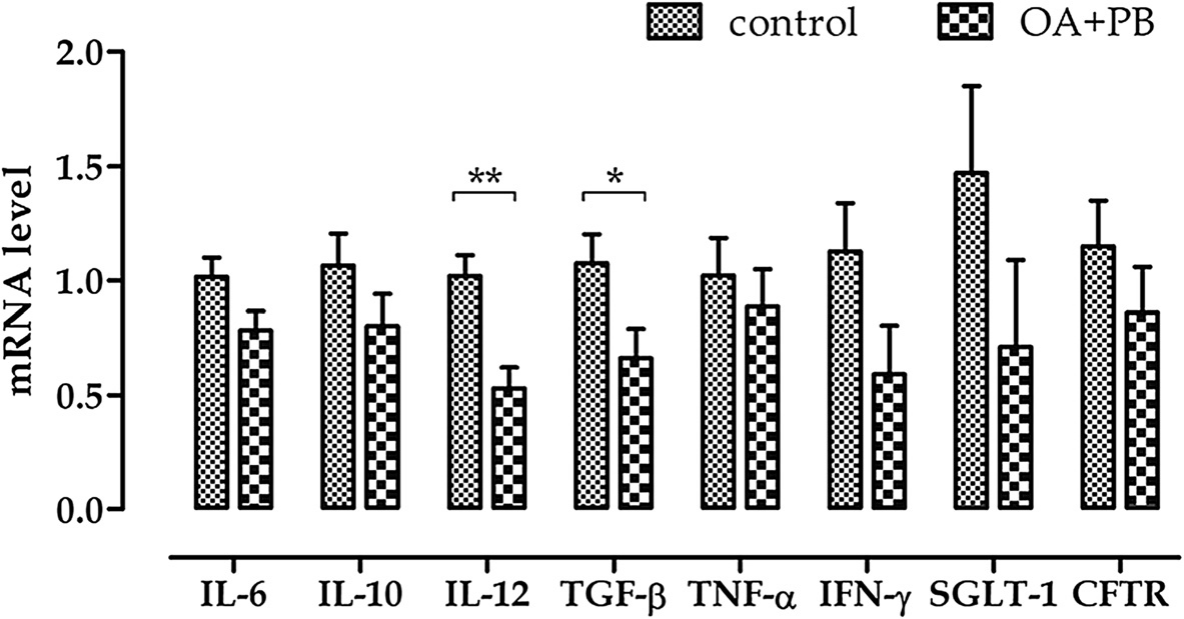
Gene expression of inflammatory cytokines, SGLT-1 and CFTR in ileal mucosa. Values are least square means (*n* = 6) and SEM represented by vertical bars. Within a gene, columns with symbols are different: * = *P* < 0.05; ** = *P* < 0.01. A modification of the 2^–ΔΔC^_T_ method was used to analyze the relative expression (fold changes), calculated relative to the control group (control), [17]. Control = basal diet; OA + PB = basal diet + mixture of organic acids and pure botanicals at 5 g/kg.

**Figure 3:**
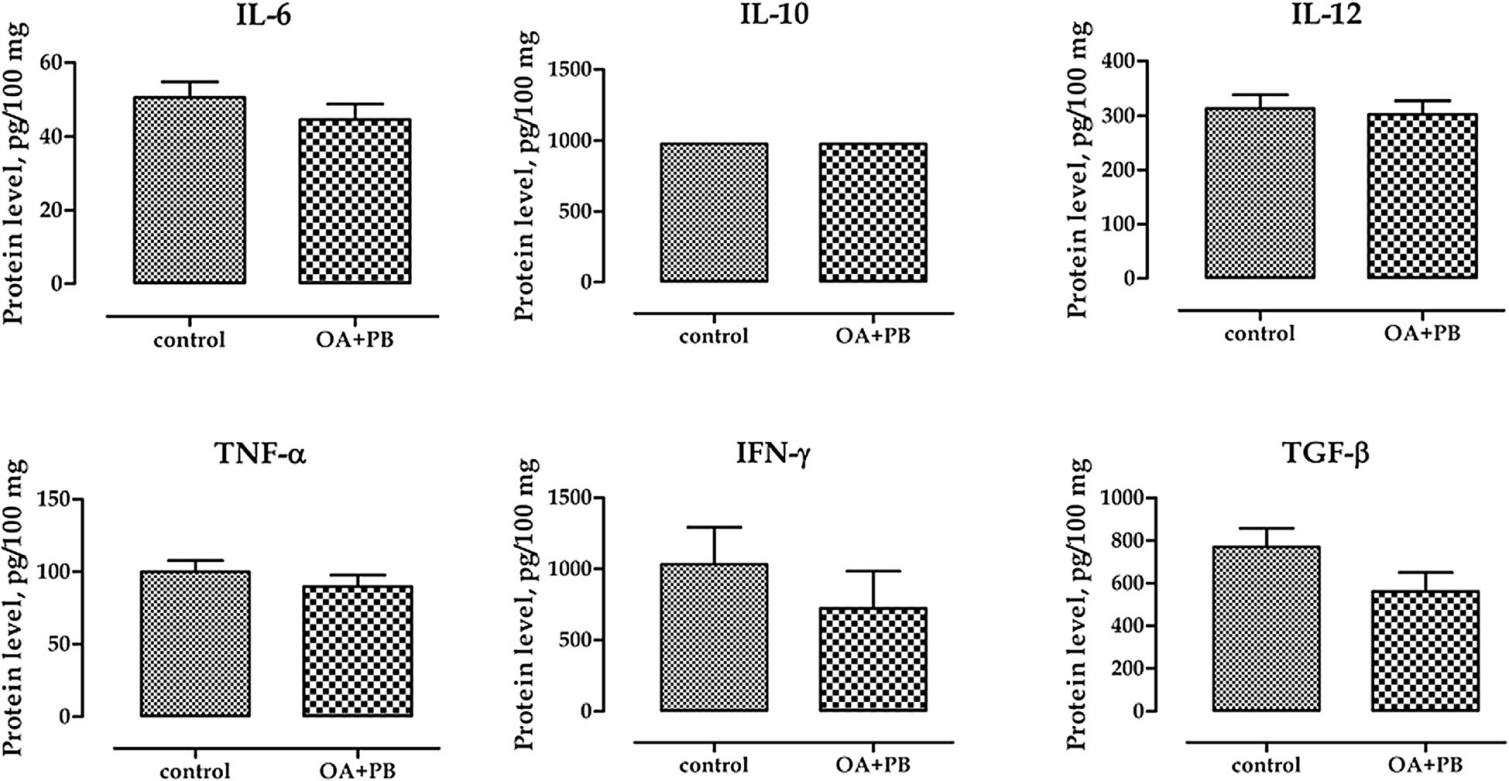
Protein levels of inflammatory cytokines in ileal mucosa. Values are least square means (*n* = 6) and SEM represented by vertical bars. Data refer to picograms of cytokine per 100 milligrams of tissue (pg/100 mg) measured using ELISA method. Control = basal diet; OA + PB = basal diet + mixture of organic acids and pure botanicals at 5 g/kg.

Figure 2 shows mRNA levels of inflammatory cytokines, SGLT-1 and CFTR in ileal mucosa. The group treated with OA + PB mixture had significantly lower mRNA level of TGF-β and IL-12 (*P* = 0.04 and *P* = 0.003, respectively), and tended to have a reduced mRNA of IL-6 and IFN-γ (*P* = 0.07 and *P* = 0.10, respectively). SGLT-1 and CFTR mRNA were not affected by the treatment with OA + PB. Protein levels of inflammatory cytokines in ileal mucosa are presented in Figure 3. Compared with control group, OA + PB group had tended to have lower TGF-β level (*P* = 0.10), whereas no effects were observed in IL-6, IL-10, IL-12, IFN-γ or TNF-α protein content.

**Figure 4:**
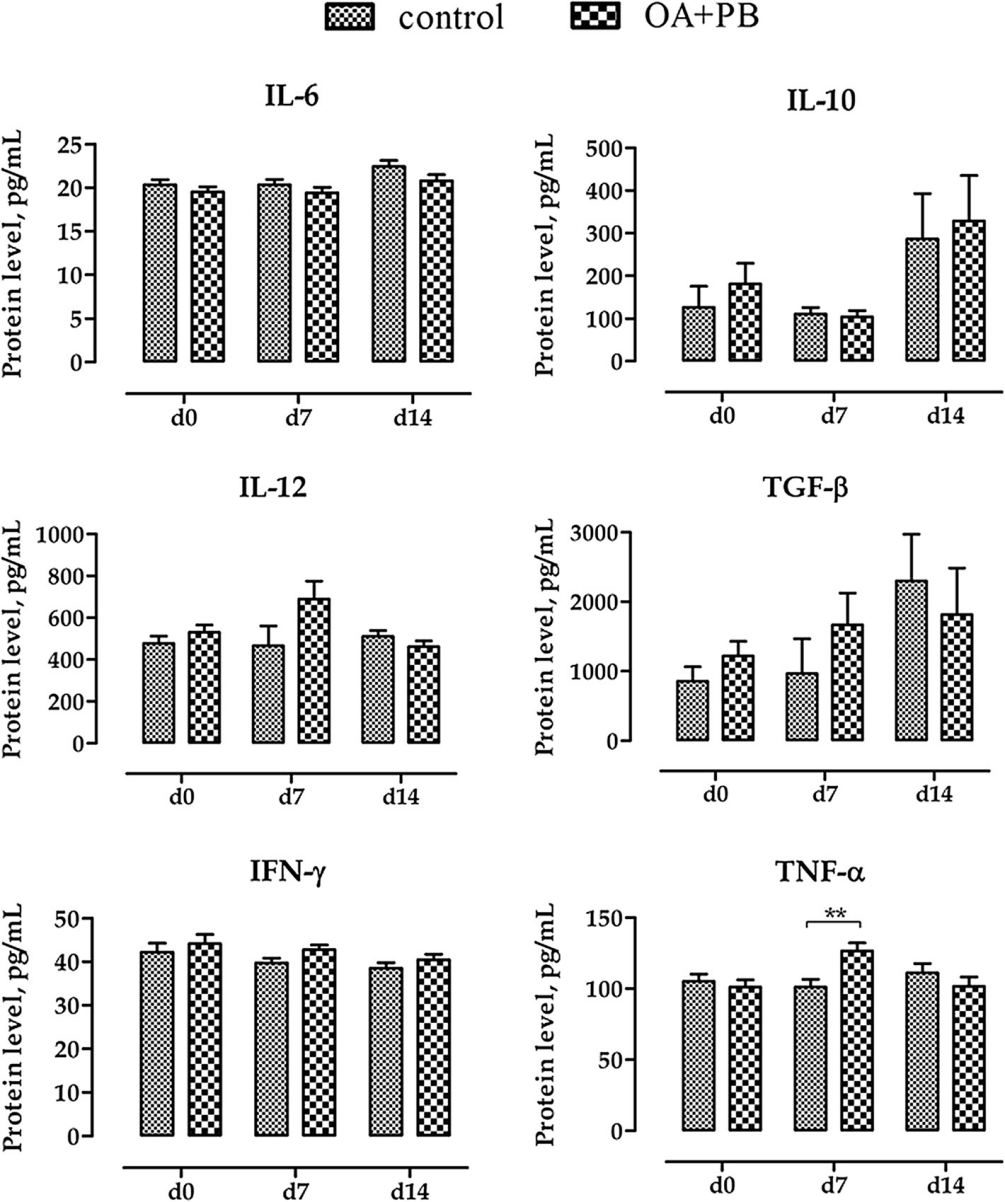
Protein levels of inflammatory cytokines in serum at d0, d7, and d14. Values are least square means (*n* = 6) and SEM represented by vertical bars. Within a time-point (d0, d7, or d14) columns with symbols are different: * = *P* < 0.05; ** = *P* < 0.01. Data refer to picograms of cytokine per milliliter of serum (pg/ml) measured using ELISA method. Control = basal diet; OA + PB = basal diet + mixture of organic acids and pure botanicals at 5 g/kg.

Figure 4 shows protein levels of inflammatory cytokines in sera samples measured at d0, d7 and d14 of the study. At d0 no differences were observed. At d7 pigs fed with OA + PB had significantly higher TNF-α (*P* < 0.01) and tended to have higher IL-12 and IFN-γ compared with controls (*P* = 0.10 and *P* = 0.08, respectively). At d14 serum cytokines were not affected by the treatment.

#### In vitro assessment on Caco-2 cells

**Figure 5:**
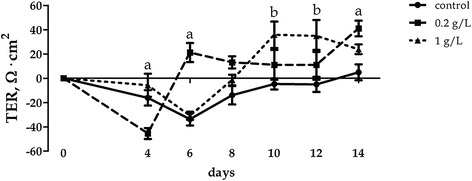
Trans-epithelial resistance of Caco-2 cells grown with 0.2 g/l or 1 g/l of OA + PB. Data in the graph are means (*n* = 7) and SEM represented by vertical bars. Superscript “a” means that at each time point 0.2 g/l was different from control, whereas “b” means that 1 g/l was different from control.

**Figure 6:**
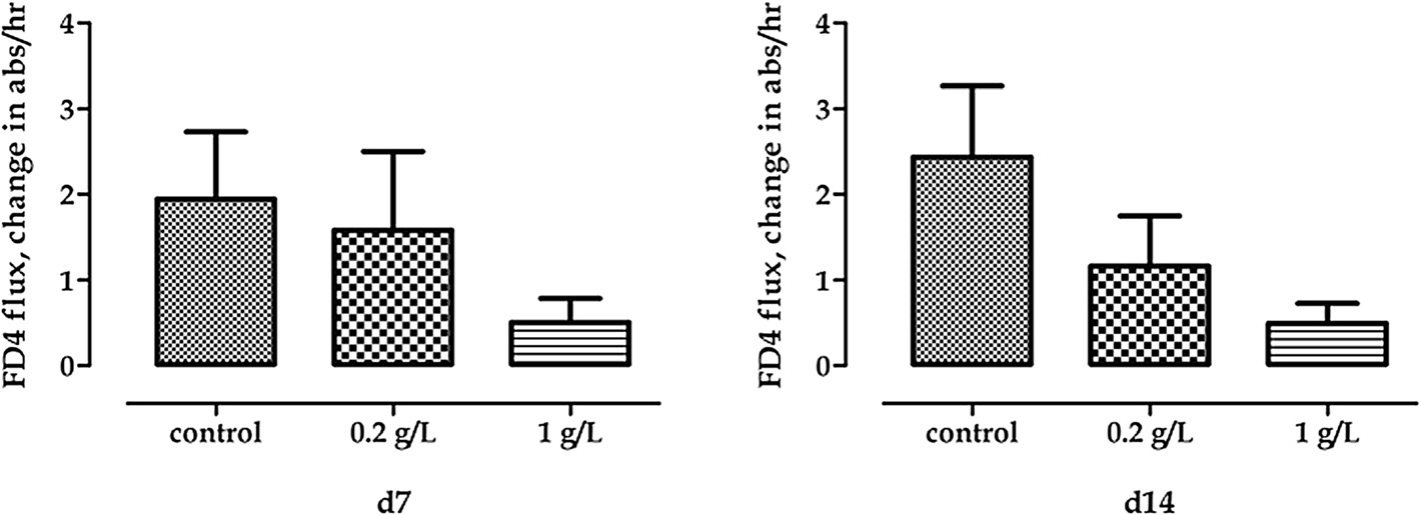
FD4 flux change of Caco-2 cells grown with 0.2 g/l or 1 g/l of OA + PB. Values are means (*n* = 7) and SEM represented by vertical bars.

Trans epithelial resistance was increased by the supplementation of OA+PB at 0.2 g/L at 4, 6, and 14 days of the experiment, whereas 1 g/l did increase TER at 10 and 12 days compared with control (*P* < 0.05; Figure 5). FD4 flux was not significantly affected though a strong trend toward decrease was observed both at 7 and 14 days (*P* = 0.10 and *P* = 0.09, respectively; Figure 6).

## Discussion

Organic acids and botanicals in pig husbandry have been widely used in association to or partial substitution of antibiotic growth promoters due to their acidifying and antimicrobial properties [8,19,20]. Previous studies from our research group showed that microencapsulation of organic acids and pure botanicals in a lipid matrix slowly releases these compounds in the gut of the pig until reaching the distal part of the intestine, where they modulate the microbial ecosystem by decreasing coliform populations [21]. Weaning, in addition to the morphological and functional changes that impair the intestinal functions, is associated with up-regulation of pro-inflammatory cytokines [22], and previous studies have shown a consistent impairment of the intestinal barrier and of the mucosal immune response to pathogenic challenges with both transient and long-term consequences [23,24]. In this study piglets receiving the diet supplemented with OA + PB for 2 weeks post weaning had better daily growth and greater body weight than control animals and this result is consistent with previous findings from our research group where piglets fed with the same OA + PB had an improved growth performance and intestinal metabolism compared with a control diet [11]. In this study, though, we did want to explore more in depth the reasons at the basis of this enhanced growth and we therefore measured the effects of the treatment with OA + PB on the intestinal integrity by Ussing chamber analysis. The treatment did not impact the TER, there was a slight decrease in FD4 flux, although not-significant, in the jejunum of treated animals, which was accompanied by a decrease in basal *I*_SC_ in both jejunum and ileum compared with control animals. A reduced *I*_SC_ might be the reflection of either a decrease in anion secretion (e.g. Cl^−^, HCO_3_^−^) or an enhancement of secretion of cations such as K^+^. Without using isotopic ion flux measurement it is difficult to understand the precise nature of this *I*_SC_ response and for this reason we measured the ileal gene expression of the cystic fibrosis transmembrane conductance regulator (CFTR), which is an apical anion channel responsible for the cAMP-dependent efflux of Cl^−^ ions into the lumen [25], and of sodium-dependent glucose transporter (SGLT-1). Despite no major effect on CFTR, we observed a numerical decrease of SGLT-1 expression that could be reflective of a higher Na^+^ reabsorption mediated by an higher amount of transporter by a negative feedback regulation mechanism. The epithelial permeability is continuously regulated by several physiological, bacterial, and dietary stimuli, among which inflammatory cytokines are regarded as major mediators [26]. Pro-inflammatory cytokines such as IFN-γ, TNF-α, IL-12 and IL-1β induce the para-cellular permeation to luminal compounds by inducing disruption of tight junctions, whereas anti-inflammatory cytokines instead, including IL-10 and TGF-β, tend to protect the epithelial integrity [27]. In this experiment the treatment with OA + PB modulated the inflammatory status in the ileum mucosa: compared with the control group, mRNA tended to be reduced for IFN-γ and IL-6 and was significantly reduced for IL-12 and TGF-β. This reduction of mRNA levels, which was not paralleled by protein levels though, may be the mechanism at the basis of a tighter epithelium. It seems, therefore, that OA + PB positively modulated the inflammatory stress in the intestinal epithelium by reducing pro-inflammatory stimulus, rather than enhancing the anti-inflammatory response. In this view, the reduction of both gene and protein expression of the anti-inflammatory cytokine TGF-β can be considered as a consequence of the reduced pro-inflammatory milieu in the intestinal mucosa.

Treatment with OA + PB also influenced the systemic immune response of the piglets as indicated by the inflammatory cytokine levels in the serum: after the first week of treatment, the OA + PB group had increased circulating levels of TNF-α and IFN-γ, as well as numerically higher IL-12, compared to control group. This result might appear surprising as these three cytokines are pro-inflammatory, but no adverse effects on the piglets health were observed in the present study, neither immuno-stimulatory responses have been ascribed to organic acids and botanicals in previous studies. One possible explanation may depend on the role of IL-12 as a coordinator of the innate and adaptive immune responses. As a such, IL-12 stimulates the production of IFN-γ by natural killer cells, T cells, macrophages and dendritic cells, as well as the differentiation of IFN-γ-producing T helper 1 cells, therefore regulating the development of the adaptive immunity [28]. In this view, in piglets fed OA + PB after the first week, IL-12 may have driven the transient over-expression of circulating pro-inflammatory cytokines, thus suggesting a more rapid development of the immune response induced by OA + PB. This immuno-stimulation induced by OA + PB was transient and disappeared early after weaning, as shown by the results at the end of the study, when no differences in the inflammatory cytokines levels were observed.

The existing symbiosis between the host and the microbiota is mediated by the host immune system. At the gut level a large population of bacteria exerts a consistent immunological pressure which usually results in sub-chronic inflammation also in physiological condition. This process is further exacerbated at weaning when acute inflammation may occur as a result of a complex cascade of events which often ends in microflora imbalances in favors of harmful species. Organic acids and botanicals have proven antimicrobial properties both *in vitro* and *in vivo* [29] and their use in pig diets has always been justified by their capacity to control harmful pathogens and unhealthy microflora in the gut. Nevertheless, it remains to be unveiled whether these substances may exert a direct effect –non-microflora-mediated– on the mucosa. Therefore, as we wanted to “knock-out” the possible role of the microbiota, we exposed Caco-2 cell cultures to OA + PB for 2 weeks. Caco-2 cells are in fact often used as a model to study the small intestine as they are capable of exhibiting structural and functional differentiation patterns characteristic of mature enterocytes [30], and the increased TER measured over time indeed proves the direct role of OA + PB in ameliorating epithelial integrity via a microflora -independent pathway. The exact mechanism by which OA + PB would regulate the epithelial integrity is beyond the objective of this study although some explanation might be derived by the multiple properties and biological functions of these compounds. More specifically, sorbic acid has been recently demonstrated to improve the growth of pigs at weaning by regulating IGF gene expression and secretion [31], and citric acid, a TCA cycle intermediate, plays an important role as energy substrate for the cell. On the other end, the use of plant extracts, such as monoterpens and aldehydes, has been associated with a generally improved gut equilibrium [8] as these molecules can alter the expression of genes related to the activation of the immune response and the expression of genes related to integrity of membranes [32].

## Conclusions

In conclusion, OA + PB have the potential to induce a more rapid maturation of the intestinal mucosa by decreasing the local and systemic inflammatory pressure, ultimately resulting in a less permeable intestine, and eventually improving the growth of piglets prematurely weaned. Moreover, in this study we also elucidated another mechanism of action of OA + PB, beyond the anti-microbial one, which is host-mediated and microflora-independent, as observed in the cell culture study in the absence of any microbial stimuli.

## Abbreviations

OA + PB: Organic acids and pure botanicals
ADG: Average daily gain
PD: Potential difference
TER: Trans-epithelial electrical resistance
*I*_SC_: Short-circuit current
IFN-γ: Interferon- γ
TGF-β: Transforming growth factor-β
TNF-α: Tumor necrosis factor- α
IL-6: Interleukin-6
IL-10: Interleukin-10
IL-12: Interleukin-12
RPL35: Ribosomal protein L35
RPL4: Ribosomal protein L4
GOI: Gene of interest
HKs: Housekeeping genes
SGLT-1: Sodium/glucose co-transporter 1
CFTR: Cystic fibrosis transmembrane conductance regulator
BW: Body weight
